# The role of exercise induced capillarization adaptations in skeletal muscle aging: a systematic review

**DOI:** 10.3389/fphys.2025.1681184

**Published:** 2025-09-26

**Authors:** Yi Ding, Qiliang Wan, Jae Cheol Kim, Wenduo Liu, Weiping Ji

**Affiliations:** ^1^ Department of Physical Education and Military Training, Zhejiang Ocean University, Zhoushan, China; ^2^ Department of Sports Science, College of Natural Science, Jeonbuk National University, Jeonju, Republic of Korea

**Keywords:** skeletal muscle aging, capillarization, exercise adaptation, aerobic exercise, resistance training

## Abstract

**Background:**

Skeletal muscle aging is often accompanied by capillary rarefaction, which limits the effective delivery and distribution of hormones, nutrients, and growth factors within skeletal muscle. Furthermore, exercise is widely regarded as having the potential to improve microcirculation and delay skeletal muscle aging. This review aims to explore exercise-induced improvements in capillarization and related adaptations to mitigate the adverse changes that occur during the aging process of skeletal muscle.

**Methods:**

This systematic review was conducted in accordance with the PRISMA guidelines and registered in the PROSPERO database under the identifier CRD420251055873. Studies involving exercise interventions in older adults were included, with the requirement that at least one original outcome related to skeletal muscle capillarization was reported. Articles were rigorously screened based on the PICOS criteria, and the quality of the included studies was assessed.

**Results:**

Studies have shown that older adults still possess the capacity to improve skeletal muscle capillarization through exercise. Moderate-intensity aerobic exercise not only significantly enhances the level of capillarization but also induces effects that can be maintained even after cessation of training. Capillarization adaptations induced by resistance training exhibit marked inter-individual variability, which is primarily determined by each individual’s baseline level of capillarization, thereby resulting in distinct patterns of adaptation. The studies also revealed that the regulation of capillarization depends on the synergistic action of VEGF and eNOS, and that different types of exercise may elicit adaptations through distinct molecular pathways.

**Conclusion:**

During the aging process, exercise-induced improvements in capillarization can enhance nutrient delivery, metabolic efficiency, and regenerative capacity in skeletal muscle. To some extent, these adaptations help suppress degenerative changes in muscle function and provide a targeted foundation for anti-aging intervention strategies.

## 1 Introduction

In recent years, with the increasing depth of research on skeletal muscle aging, the adaptive changes in skeletal muscle capillarization have gradually attracted growing attention. As critical bridges between the circulatory system and muscle fibers, capillaries—through their density and distribution characteristics—constitute what is referred to as capillarization (Hellsten and Gliemann, 2024). Capillarization not only determines the blood flow contact surface surrounding muscle fibers but also influences the efficiency of oxygen and amino acid delivery, as well as the removal of metabolic waste products (Payne and Bearden, 2006; Poole et al., 2021; Poole and Musch, 2023). However, with advancing age, the level of capillarization around skeletal muscle fibers shows a declining trend (Croley et al., 2005), leading to a series of degenerative changes such as reduced efficiency of oxygen metabolism in skeletal muscle and limitations in muscle protein synthesis (Groen et al., 2014; Prior et al., 2016).

It is noteworthy that capillary rarefaction is not merely a concomitant phenomenon of skeletal muscle aging, but may also serve as a critical upstream mechanism driving structural and functional degeneration of skeletal muscle (Landers-Ramos and Prior, 2018). A study on older adults with systemic sclerosis found that capillary rarefaction may occur prior to the reduction of skeletal muscle mass (Paolino et al., 2020), suggesting that capillary impairment could serve as an early factor influencing degenerative changes in muscle. Therefore, in the process of skeletal muscle aging, the regulation of muscle capillarization may represent an earlier and more fundamental target for intervention. Nonetheless, current research on the underlying mechanisms in this area remains limited, particularly with respect to in-depth investigations into how the regulation of capillarization levels may suppress skeletal muscle aging.

As one of the most widely practiced health-promoting behaviors, exercise has been proven to effectively delay aging-related functional decline in skeletal muscle (Wang et al., 2024; Liu et al., 2025). A substantial body of research indicates that exercise interventions not only improve muscle mass and strength in older adults but also show great potential in enhancing skeletal muscle capillarization (Gavin et al., 2015; Moro et al., 2019). An expanded capillary network provides a broader contact surface with muscle fibers (Betz et al., 2024), not only enhancing the exchange efficiency of oxygen and metabolic substrates (Gliemann et al., 2021), but also creating a favorable biochemical foundation for protein synthesis (Moore et al., 2018), and promoting the activation and proliferation of satellite cells (SCs) (Snijders et al., 2019), thereby facilitating the adaptive remodeling of muscle.

In summary, this systematic review aims to clarify the characteristics of skeletal muscle capillarization changes during aging and their impact on muscle function, further highlighting its potential value as an early intervention target. It also seeks to elucidate the underlying mechanisms and therapeutic significance of exercise-induced regulation of skeletal muscle capillarization in mitigating the aging process. Ultimately, the goal is to provide a theoretical foundation for optimizing exercise strategies against the negative changes of aging and to support future research into the regulatory mechanisms of capillarization involved in skeletal muscle aging.

## 2 Methods

### 2.1 Protocol and registration

This systematic review was conducted in accordance with the Preferred Reporting Items for Systematic Reviews and Meta-Analyses (PRISMA) guidelines (Page et al., 2021), and was registered in the PROSPERO database under the identifier CRD420251055873.

### 2.2 Literature search strategy

This study conducted a systematic search in accordance with the PRISMA statement. Literature searches were performed in the PubMed, Web of Science, Scopus, and Embase databases, covering the period from 2000 to March 2025. The search was limited to articles published in English, and only original research studies were included. Review articles, conference abstracts, case reports, and other non-original research types were excluded. Authors Y.D. and Q.W. independently screened the relevant articles based on the inclusion criteria. Any discrepancies regarding inclusion or exclusion were resolved through discussion with a third author (W.L.). The search terms used are detailed in Supplementary Material.

In this review, the starting point for literature retrieval was set at the year 2000 based on the following considerations: (1) The primary aim of this review was to summarize the most recent evidence regarding the effects of exercise on skeletal muscle capillarization in older adults, thereby enhancing the relevance of the findings for current exercise practice and healthy aging; (2) A marked increase in the number of related studies has been observed since 2000, with greater standardization in intervention design, methodological description, and outcome reporting, which improves the reproducibility of research findings; (3) According to the inclusion criteria, eligible studies published before 2000 were extremely limited; Therefore, restricting the search to studies published after 2000 does not compromise the validity of the review’s conclusions.

### 2.3 Inclusion and exclusion criteria

This systematic review established the inclusion and exclusion criteria based on the PICOS framework. Included studies involved participants who were clearly in the older age stage (≥60 years) or approaching older age (55–59 years); the intervention consisted of exercise training, including aerobic exercise, resistance training, or combined modalities; primary outcomes were required to include at least one indicator related to skeletal muscle capillarization; and the study design had to be either experimental or observational original research.

Reviews, conference abstracts, case reports, studies lacking original data or with unclear exercise interventions, and studies involving only non-aging-related populations were excluded.

### 2.4 Data extraction

Data were extracted from the selected studies using a pre-designed Excel 2021 spreadsheet. The extracted information included: basic study information (first author, year of publication), study design type, participant characteristics (age, sex, sample size), intervention details (type, frequency, intensity, and duration of exercise), capillarization indices, and main findings. Two authors (Y.D. and Q.W.) independently performed the data extraction, and any discrepancies were resolved through discussion with a third author (W.L.).

### 2.5 Assessment of study quality

To ensure the reliability of the included studies, this systematic review assessed study quality using the Mixed Methods Appraisal Tool (MMAT). The MMAT is a widely used tool that facilitates the quality assessment of studies with diverse designs (Hong et al., 2018). The MMAT uses design-specific questions to assess the appropriateness of study design, data, and analytical methods across five domains of study quality (Pace et al., 2012), with each domain rated as “Yes,” “No,” or “Can’t tell.” The number of “Yes” ratings was summed to generate a total score. Scores of 0–2 were considered low quality, 3–4 as moderate quality, and 5 as high quality. All quality assessments were independently conducted by two researchers (Y.D. and Q.W.). Any discrepancies in the evaluation were resolved through discussion, and if necessary, determined by a third researcher (W.L.).

### 2.6 Analytical methods

Due to the high heterogeneity among the studies, a meta-analysis was not performed. The results were presented using a narrative synthesis approach.

## 3 Results

### 3.1 Search results and study selection

**FIGURE 1:**
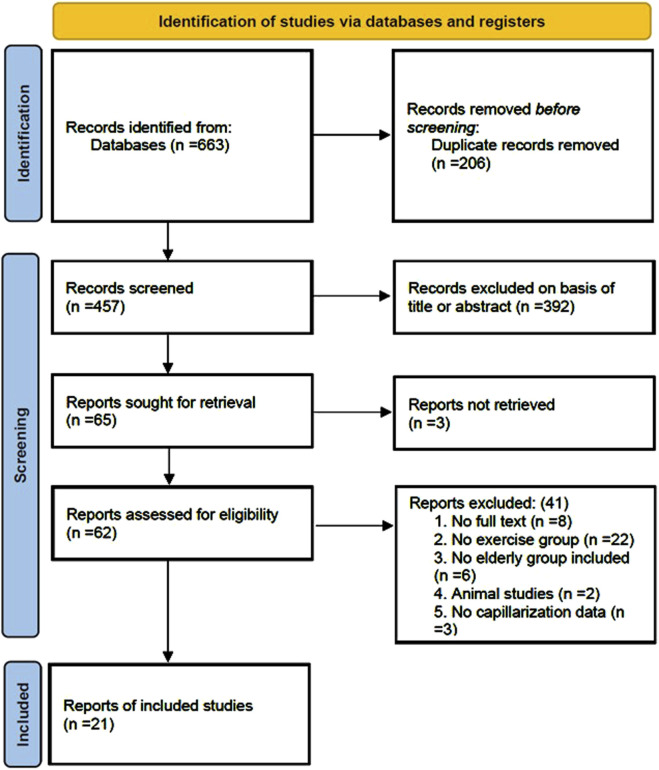
Flow diagram of study selection.

A total of 663 articles were retrieved from the four databases: PubMed (106), Web of Science (134), Scopus (222), and Embase (201). After removing 206 duplicate articles, the titles and abstracts of the remaining articles were screened, and 392 articles were excluded, including reviews, conference abstracts, and case reports. The remaining 65 articles underwent full-text assessment. During the download process, 3 articles could not be retrieved. After thorough examination of the remaining 62 articles, 8 were excluded due to the lack of full text. In addition, 22 articles did not include an exercise group and only conducted correlation analyses between capillarization indices and skeletal muscle aging. Six articles did not include an elderly group as study participants, and two were animal studies, all of which did not meet the inclusion criteria. Three articles did not report capillarization levels in the elderly population. After full-text screening, 41 articles were excluded. Ultimately, 21 articles were included in the analysis (Figure 1).

### 3.2 Study characteristics

A total of 21 studies were included, and their characteristics are presented in Table 1. These studies were conducted between 2000 and 2025 and all involved older adults (aged 55–82 years), with some being comparative studies between older and younger individuals. All studies reported capillarization indices in elderly participants and their responses to exercise, which served as the primary focus of analysis in this review. The intervention modalities included aerobic exercise, combined aerobic and resistance training, high-intensity interval training (HIIT), and traditional resistance training, demonstrating a diverse range of exercise types. The intervention duration ranged from a single exercise session to a maximum of 24 weeks, with 8-week and 12-week programs being the most common. The training intensity was mostly moderate to high. The definition of training intensity was based on descriptions provided in the included studies. To ensure consistency, aerobic exercise intensity was expressed as a percentage of maximal oxygen uptake (%VO_2_max), while resistance training intensity was expressed as a percentage of one-repetition maximum (%1RM) (Bull et al., 2020; Izquierdo et al., 2021). Accordingly, exercise intensity was categorized into three levels: low intensity (<40% VO_2_max or <50% 1RM), moderate intensity (40%–70% VO_2_max or 50%–75% 1RM), and high intensity (>70% VO_2_max or >75% 1RM).

**TABLE 1:** Summary of included study characteristics.

Study	Age	Gender	Exercise type	Intensity	Duration	Capillarization	Comparison extracted	Main finding
Betz et al. (2024)	72 ± 6	Mix	RT	High	Acute	CC↔ C/Fi↔ CFPE↔	Pre vs. Post	Capillarization positively associated with post-exercise blood flow
Charles et al. (2006)	74 ± 4	Male	AE + RT	High	14 W	LC/PF↑ Kf↑	Pre vs. Post	LC/PF was significantly correlated with Kf
Croley et al. (2005)	70 ± 8	Female	AE	Low	Acute	CD↔ CFPE↔	Pre vs. Post	VEGF↑ C/Fi significantly associated with FCSA
Gavin et al. (2015)	65 ± 2	Female	AE	Moderate	8W	CD↑ CC↑ C:F↑ CFPE↑	Pre vs. Post	VEGF↑ Blood flow↑
Gavin et al. (2007)	64 ± 2	Male	AE	Moderate	8W	CD↑ CC↑ C:F↑	Pre vs. Post	VEGF↑
Gliemann et al. (2014)	65 ± 1	Male	AE	High	8W	C:F↑ CD↑	Exe vs. Con	VEGF↑
Gliemann et al. (2021)	61 ± 4	Female	PA	Highly active	Long-term	C:F↑ CD↑	High activity vs. Moderate activity vs. Inactive	VEGF↑ Complex Ⅰ↑ Complex Ⅱ↑ Complex Ⅴ↑
Iversen et al. (2011)	71.3 ± 4	Mix	AE	Moderately active	Long-term	C:F↑	Exe vs. Con	VEGF↑
Leuchtmann et al. (2020)	66.5 ± 3.8	Male	HIIT RT	High Moderate	12W 12W	C:F↑ CC↑	Exe vs. Con	HIIT: CFPE↑ Shared factor↓
McKendry et al. (2020)	67.1 ± 6.4	Male	AE	Moderately active	Long-term	CC↑ C:F↑ CFPE↑	Exe vs. Con	VO_2_max approaching the level of young adults
Moore et al. (2018)	71 ± 5	Male	RT	Low	2W	CD↔ C:F↑ C/Fi↑	Exe vs. Con	Protein synthesis↑ C/Fi moderately correlated with protein synthesis
Moro et al. (2019)	71.1 ± 4.3	Mix	RT	Moderate	12W	CFPE-L Group C/Fi↑ CFPE↑	High vs. Low baseline CFPE	Satellite cells activity↑ Satellite cells activity positively correlated with CFPE
Mortensen et al. (2019)	57 ± 9 53 ± 7	Mix	END HIIT	Moderate High	11W	END: C:F↑ HIIT: C:F↔ CD↔	Exe vs. Con	eNOS protein expression increased progressively with training in the HIIT group
Murias et al. (2011)	69 ± 7	Male	AE	Moderate–High	12W	CD↑ CC↑ C/Fi↑ CFPE↑	Pre vs. Post	Citrate synthase activity↑ VO_2_max↑
Olsen et al. (2020)	64 ± 4.2	Female	AE	High	8W	CD↔ CC↔ C:F↔ CFPE↔	Pre vs. Post	VEGF↑ TSP-1↓ VEGF/TSP-1↑
Pollock et al. (2018)	55-79	Mix	AE	Moderately active	Long-term	CD↓with age in males	None	CD, C:F, and CC significantly correlated with O_2_ uptake kinetics
Prior et al. (2015)	65 ± 3	Mix	AE	High	6M	CD↑	Pre vs. Post	Insulin sensitivity↑
Ryan et al. (2006)	65 ± 4	Male	AE	Moderate	Acute	CC↔ CD↔ C:F↔	Pre vs. Post	VEGF↑
Snijders et al. (2019)	74 ± 8	Male	RT + HIIT	High	12W	C/Fi↑ CC↑	Pre vs. Post	Satellite cell–capillary distance ↓ C/Fi ↑ associated with satellite cell number↑ CC ↑ closely associated with satellite cell activation
Snijders et al. (2017)	71 ± 1	Male	RT	High	24W	CFPE-L Group C/Fi↑	Pre vs. Post	Only individuals with higher baseline capillarization showed significant increases in muscle fiber size and satellite cell number
Verdijk et al. (2016)	72 ± 1	Male	RT	Moderate High	12W	CC↑ CD↑ C/Fi↑ CFPE↑	Pre vs. Post	Greater capillarization is associated with increased satellite cell abundance

Abbreviations: AE, aerobic exercise; RT, resistance training; PA, physical activity; HIIT, high-intensity interval training; END, endurance training. Pre vs. Post, comparison before and after exercise intervention; Exe vs. Con, comparison between exercise group and control group.

In addition, observational studies involving long-term exercisers were included, all of whom had over 10 years of exercise experience. All studies collected the vastus lateralis muscle as the site of analysis, providing a consistent basis for standardized measurement of capillarization and skeletal muscle function. To standardize terminology and facilitate understanding, this review summarizes the abbreviations and definitions of key capillarization indices (Table 2).

**TABLE 2:** Abbreviations and definitions of capillarization indices.

Abbreviation	Full term	Definition
CD	Capillary Density	Number of capillaries per unit area
CC	Capillary Contacts	Total number of capillaries in direct contact with muscle fibers
CFPE	Capillary-to-Fiber Perimeter Exchange	Number of capillary contacts per unit of fiber perimeter
C:F	Capillary-to-Fiber Ratio	Average number of capillaries per muscle fiber
C/Fi	Capillaries per Individual Fiber	Number of capillaries contacting a specific muscle fiber

### 3.3 Study quality

Among the included studies, 15 were non-randomized intervention studies, 4 were cross-sectional studies, and 2 were randomized controlled trials. Details of study quality are presented in Supplementary Table. While some studies demonstrated strong methodological rigor, others exhibited notable limitations.

**FIGURE 2:**
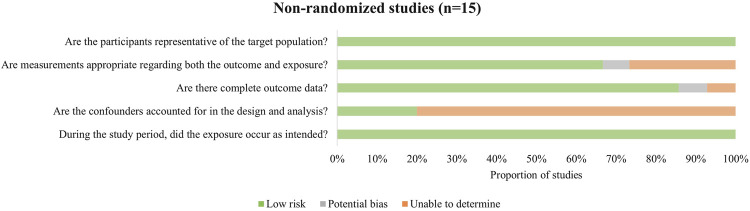
Methodological quality assessment of non-randomized studies.

Non-randomized intervention studies constituted the majority of included studies in this review (Figure 2). The main potential sources of bias were insufficient control of confounding factors and lack of sample representativeness. Among these studies, one did not clearly report how missing data were handled, and another did not provide detailed information on outcome measurement methods. Additionally, a few studies did not fully report the specific results of key outcome indices.

**FIGURE 3:**
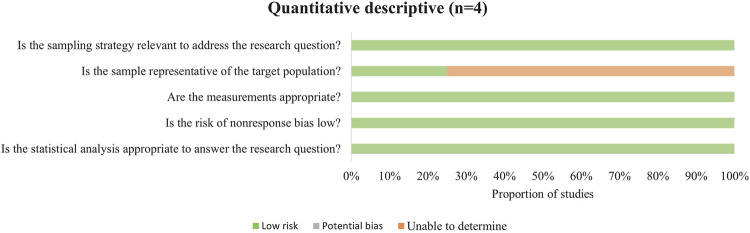
Methodological quality assessment of cross-sectional studies.

In the cross-sectional studies (Figure 3), three studies involved populations that lacked representativeness, which limited the generalizability of their findings.

**FIGURE 4:**
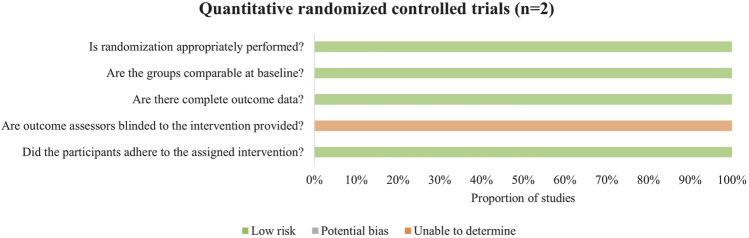
Methodological quality assessment of randomized controlled trials.

Both randomized controlled trials did not clearly describe the randomization procedures (Figure 4). Additionally, due to the nature of exercise interventions, blinding of outcome assessors was not implemented in the studies.

Overall, the study quality ranged from moderate to high, indicating generally acceptable methodological standards; however, the limitations should be carefully considered when interpreting and applying the results.

### 3.4 Regulatory effects of exercise on skeletal muscle capillarization

3 studies reported that 8 weeks of moderate-intensity aerobic exercise could enhance skeletal muscle capillary CD, CC, C:F, and CFPE index (Gavin et al., 2007; 2015; Gliemann et al., 2014). No changes in capillary parameters were observed during 8 weeks of high-intensity aerobic exercise (Olsen et al., 2020). Further studies showed that 11–12 weeks of moderate-intensity aerobic exercise had the same enhancing effect as 8 weeks of moderate-intensity aerobic exercise, effectively improving skeletal muscle capillarization and significantly increasing CD, CC, C:F, and CFPE (Murias et al., 2011; Mortensen et al., 2019). Longer-term moderate-to-high intensity aerobic exercise for 6 months increased CD by 15%, and this elevated level was maintained even during the 2-week detraining period after the end of training (Prior et al., 2015). In addition, in groups engaged in lifelong aerobic exercise, the overall level of capillarization was found to be significantly higher than in sedentary individuals (Iversen et al., 2011; McKendry et al., 2020; Gliemann et al., 2021). Compared with the capillary adaptations induced by long-term aerobic exercise interventions, whether a single bout of aerobic exercise can affect changes in capillary-related indices is also a focus of the present study. In two reports investigating a single bout of aerobic exercise, no changes in capillarization indices were observed (Croley et al., 2005; Ryan et al., 2006).

Similar to aerobic exercise, a single bout of resistance training does not lead to significant changes in capillarization indices. However, one study reported that a single bout of resistance training significantly increased microvascular perfusion capacity (Betz et al., 2024). Since existing aerobic exercise studies have not assessed microvascular perfusion capacity, whether it exerts a comparable effect remains unknown and requires further investigation. Two weeks of low-intensity resistance training significantly increased C:F (Moore et al., 2018). In contrast, a 12-week moderate-intensity resistance training intervention showed no changes in capillarization indices after exercise. Interestingly, when participants were grouped according to baseline CFPE index, it was found that after 12 weeks of training, the low-baseline group showed significant increases in C/Fi and CFPE (Moro et al., 2019). Notably, progressively increasing training intensity elevated CC, C/Fi, and CFPE levels in older men to values comparable to those of young adults (Verdijk et al., 2016). Another study using baseline CFPE stratification further confirmed the findings. After 24 weeks of moderate-to-high intensity resistance training, C/Fi was significantly increased in the low-initial group, and the difference from the high-initial group was reduced (Snijders et al., 2017).

Combined training protocols integrate both metabolic and mechanical stimuli and are considered to have synergistic effects on capillary structural adaptations. A 12-week combined training program of moderate-to-high intensity resistance training and HIIT showed a significant increase in C/Fi (Snijders et al., 2019). A 12-week HIIT combined with 12-week resistance training effectively increased C/Fi and CC, while CFPE showed a significant increase only in the HIIT group (Leuchtmann et al., 2020). In addition, a 14-week combined intervention of moderate-to-high intensity aerobic and resistance training increased the capillary length per fiber perimeter (LC/PF) (Charles et al., 2006). LC/PF is a structural parameter that measures the distribution of capillaries along the muscle fiber surface, reflecting the total capillary length relative to the fiber perimeter, and indirectly indicating the potential for local substrate exchange (Charifi et al., 2004). A higher LC/PF can effectively promote the uptake and utilization of nutrients by skeletal muscle, thereby enhancing metabolic responses and the potential for protein synthesis (Charles et al., 2006; Moro et al., 2019).

### 3.5 Regulation by core factors

The regulation of skeletal muscle capillarization is coordinated by various signaling molecules, among which vascular endothelial growth factor (VEGF) is one of the key regulators (Tang et al., 2004). VEGF is typically upregulated in skeletal muscle cells in response to exercise-induced hypoxia or metabolic stress (Ross et al., 2023). It plays a pivotal role in promoting the proliferation and migration of vascular endothelial cells and is recognized as one of the key regulators of capillary angiogenesis (Olfert et al., 2010). Moderate-intensity aerobic exercise has been shown to significantly enhance VEGF protein expression in the skeletal muscle of elderly individuals (Olsen et al., 2020). A resistance training study conducted in older adults also found that an 8-week intervention increased VEGF protein expression by approximately 35%, accompanied by a significant increase in the C:F ratio (Gliemann et al., 2014). In addition, VEGF expression also increases in response to a single exercise session (Croley et al., 2005; Ryan et al., 2006). A single bout of acute aerobic exercise significantly elevated VEGF protein release, which was positively correlated with the number of capillary contacts (Gavin et al., 2007). Further research has indicated that acute exercise stimulation at different time points during the training period can upregulate VEGF expression (Gavin et al., 2015). It is noteworthy that individuals who engage in long-term aerobic exercise have resting VEGF protein levels that are 2.3 times higher than those in non-exercising individuals (Iversen et al., 2011). Moreover, maintaining a high level of physical activity over the long term can also induce elevated VEGF expression, with significantly higher levels observed compared to less active individuals (Gliemann et al., 2021). However, the regulation of skeletal muscle capillarization is not solely dependent on VEGF, and endothelial nitric oxide synthase (eNOS) also plays an important role. eNOS promotes angiogenesis by enhancing vasodilation and shear stress–mediated signaling (Milkiewicz et al., 2005). One study reported that eNOS protein content progressively increased during HIIT, whereas VEGF did not show significant changes under the same condition (Mortensen et al., 2019). This finding suggests that different exercise modalities may preferentially activate distinct molecular pathways. Under HIIT, the generation of stronger shear stress markedly induces eNOS activation. Overall, VEGF and eNOS together constitute the core regulatory factors of exercise-induced capillary adaptations.

### 3.6 The impact of exercise-induced capillarization adaptation on skeletal muscle function in older adults

Capillarization adaptations induced by aerobic exercise can significantly improve oxygen delivery capacity within skeletal muscle (Murias et al., 2011; McKendry et al., 2020), as well as enhance oxidative metabolic efficiency (Pollock et al., 2018). Moreover, the increase in capillary density resulting from long-term aerobic exercise is significantly associated with improvements in insulin sensitivity (Prior et al., 2015). Low-load resistance training enables older adults to achieve higher C/Fi values, which enhances the protein synthesis capacity of skeletal muscle following food intake (Moore et al., 2018). When baseline CFPE index are higher, the hypertrophic response of muscle fibers and the efficiency of protein synthesis after resistance training are more pronounced (Moro et al., 2019). After moderate-to-high intensity resistance training, older adults showed a significant increase in LC/PF, along with an upward trend in citrate synthase (CS) activity (Moro et al., 2019). Further analysis revealed a significant positive correlation between CS activity and LC/PF (Moro et al., 2019). Similarly, in aerobic exercise, a synergistic adaptive relationship was observed between increased capillarization and enhanced CS activity (Murias et al., 2011).

In addition, improvements in capillarization play an important role in the regulation of skeletal muscle regeneration. SCs are key stem cells responsible for maintaining muscle repair and regeneration (Pallafacchina et al., 2013). Studies have shown that increases in CC and CFPE induced by 12 weeks of high-intensity resistance training are positively correlated with the number of SCs, and the greater the capillary density in the muscle, the higher the abundance of SCs (Verdijk et al., 2016). Individuals with higher baseline CFPE levels showed a significant increase in the number of satellite cells within muscle fibers following resistance training intervention (Snijders et al., 2017). Moreover, after combined training, increases in C/Fi and CC were significantly associated with the rise in satellite cell number (Snijders et al., 2019).

## 4 Discussion

### 4.1 Molecular mechanisms underlying exercise-induced angiogenesis in skeletal muscle

The findings of this study indicate that both a single bout of acute exercise and prolonged aerobic or resistance training over several weeks can stimulate increased VEGF expression, contributing to the dynamic expansion of the capillary contact surface. Consistent with this, animal studies have reported similar findings (Hotta et al., 2018; Zmudzka et al., 2025). However, the expression level of VEGF protein in skeletal muscle is not only positively correlated with structural parameters of capillaries, but also closely associated with capillary neovascularization (Egginton, 2011). This association exists even at rest and becomes more pronounced following exercise (Croley et al., 2005; Gavin et al., 2007; Iversen et al., 2011). VEGF protein at rest helps maintain the stable distribution of capillaries around muscle fibers (Croley et al., 2005). Under exercise stimulation, especially acute exercise, VEGF mRNA expression is rapidly upregulated (Jensen et al., 2004; Iversen et al., 2011).

This change is primarily driven by local hypoxia caused by muscle contraction, which in turn activates transcription factors such as hypoxia-inducible factor 1α (HIF-1α), promoting the transcription and expression of angiogenic factors such as VEGF (Mason et al., 2007; Rodriguez-Miguelez et al., 2015). VEGF also acts in conjunction with shear stress induced by increased blood flow during exercise to activate eNOS. (Sun et al., 2002), thereby promoting the production of nitric oxide (NO) (Li et al., 2002; Green et al., 2004). As a key mediator, NO can further enhance the proliferation and migration of vascular endothelial cells (Cyr et al., 2020). The above pathways interact synergistically during exercise, collectively promoting the neovascularization and remodeling of skeletal muscle capillaries. Meanwhile, studies have found that exercise can suppress the expression of the endogenous angiogenesis inhibitor thrombospondin-1 (TSP-1), thereby further enhancing the effect of the VEGF signaling pathway and creating a microenvironment conducive to angiogenesis (Olsen et al., 2020).

In summary, the primary molecular mechanisms underlying exercise-induced angiogenesis in skeletal muscle include the hypoxia-mediated response via VEGF, shear stress-induced activation of eNOS, and the downregulation of angiogenesis inhibitors. Notably, these molecular responses remain active in older adults. Thus, such molecular-level reactions not only indicate that aging skeletal muscle retains the potential for exercise-induced angiogenesis but also lay the foundation for both structural and functional adaptations of the capillary network.

### 4.2 Enhancement of capillarization to counteract the adverse effects of skeletal muscle aging

**FIGURE 5:**
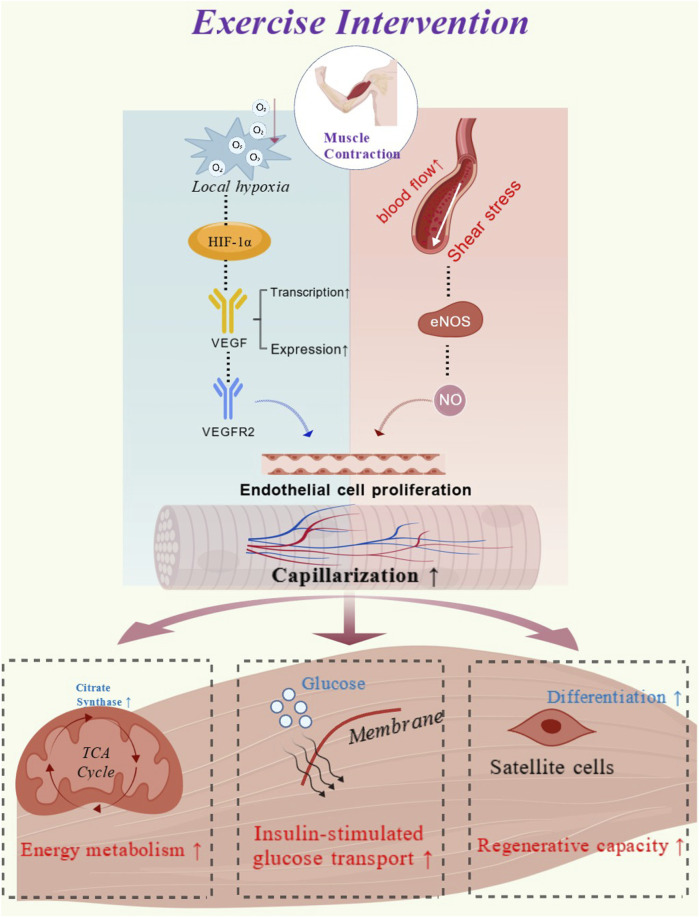
Exercise induced capillarization adaptations and their mechanistic role in promoting skeletal muscle function (created with biogdp.com. ID: GDP2025SB0M7J).

Capillaries serve as a fundamental structural basis for maintaining the stability of the muscle microenvironment (Kissane et al., 2021). Variations in capillarization levels not only determine the efficiency of energy metabolism and anabolic processes but also influence the potential for tissue regeneration and physiological adaptation (Figure 5). Studies have shown that although structural and functional adaptations of skeletal muscle capillaries exhibit heterogeneity depending on individuals’ baseline conditions and variations in exercise interventions, they generally tend to evolve toward a higher level, reflecting a positive adaptive trajectory (Degens et al., 2006; Barnouin et al., 2017). With advancing age, skeletal muscle tissue faces a range of challenges, including impaired energy metabolism, diminished protein synthesis responses, reduced insulin sensitivity, and insufficient regenerative capacity. These issues highlight the particular importance of maintaining and enhancing capillarization in older populations.

An important downstream effect of improved capillarization is the support of mitochondrial function. Structural indices such as CFPE and LC/PF reflect enhanced capacity for substrate and oxygen exchange (Charifi et al., 2004; Moro et al., 2019). Increases in these indices are closely associated with enhanced mitochondrial oxidative capacity, as evidenced by upregulation of citrate synthase activity (Larsen et al., 2012; Vigelsø et al., 2014). Therefore, an increase in CFPE is not only regarded as a marker of enhanced oxygen flux (Betz et al., 2021), but also indicates that skeletal muscle tissue receives a more adequate supply of oxygen and metabolic substrates. This, in turn, provides the necessary conditions for mitochondrial adaptation and helps sustain energy metabolism in aging skeletal muscle (Poole and Musch, 2023). Meanwhile, the capillary network also plays a central role in maintaining the anabolic capacity of skeletal muscle. One of the common characteristics of aging skeletal muscle is a diminished responsiveness to anabolic stimuli (Phillips et al., 2009; Wall et al., 2013). Increases in C:F and CC provide muscle fibers with more contact points and a denser microvascular network (Betz et al., 2024). Therefore, exercise-induced increases in capillary contact and perfusion can enhance the efficiency of amino acid delivery to muscle fibers, thereby providing the necessary structural and metabolic support for the activation of the mechanistic target of rapamycin complex 1 pathway (Sengupta et al., 2010; Ogasawara et al., 2016). Due to improved nutrient supply, the anabolic response of aging skeletal muscle is facilitated by a more favorable microenvironment (Yin et al., 2021; Yue et al., 2022).

Studies have indicated that reduced capillary density is considered a key precondition for insulin resistance, as it limits the diffusion and delivery of both glucose and insulin to muscle tissue (Hedman et al., 2000; Snijders et al., 2017). Among these indices, CD is primarily associated with the delivery of metabolic substrates and the removal of waste products (Machado et al., 2017). Studies have found that post-exercise increases in CD are positively correlated with improvements in insulin sensitivity. This adaptation may enhance insulin-mediated glucose transport and metabolic efficiency, thereby further improving metabolic health in older individuals (Prior et al., 2015). This finding is consistent with previous reports indicating that higher levels of skeletal muscle capillarization are positively associated with enhanced insulin sensitivity (Hedman et al., 2000).

The close association between the capillary network and SCs provides an additional pathway to counteract the adverse effects of aging. SCs rely on adjacent capillaries for oxygen tension and the supply of nutritional substrates (Joanisse et al., 2017), and both their number and activity are significantly increased in regions with higher capillary density (Nederveen et al., 2016; Broer et al., 2024). The number of SCs serves as a reference indicator for the progression of skeletal muscle aging (Verdijk et al., 2010). Exercise induced elevation in capillarization can enhance their activity and proliferative capacity (Thomas et al., 2022), thereby improving the repair and regeneration capacity of skeletal muscle during the aging process (Joanisse et al., 2017).

### 4.3 Exercise intervention strategies to improve skeletal muscle capillarization in older adults

Based on the mechanisms and functional roles described above, how to improve capillarization through appropriate exercise to counteract the adverse effects of aging has become a critical and practical issue in achieving healthy aging. In older adults, moderate intensity aerobic training represents a stable and effective exercise modality that can continuously induce angiogenic signaling and promote the remodeling of the capillary network, thereby providing reliable structural support for energy metabolism in skeletal muscle. The effects of resistance training exhibit inter-individual variability. Individuals with lower baseline levels of capillarization are more likely to experience improvements in capillarization indices following resistance training, whereas those with higher baseline capillarization levels tend to show significant increases in muscle fiber cross-sectional area and the number of SCs. It is noteworthy that the improvement in capillarization observed in individuals with low baseline levels primarily reflects a compensatory response driven by initial insufficiency, rather than a superior training benefit. Due to the close coupling between capillary supply and the structural and functional properties of skeletal muscle (Croley et al., 2005). Resistance training induced muscle fiber hypertrophy, particularly the increase in cross-sectional area of type II fibers, further elevates the demand for capillary supply (Snijders et al., 2017; Moro et al., 2019). In this process, the integrity of the capillary network may serve as a critical condition for mitigating the adverse effects of muscle aging through resistance training.

### 4.4 Limitations and future perspectives

This study has certain limitations. First, this review adopted a narrative synthesis approach to present the research findings rather than conducting a meta-analysis, which may limit the capacity for quantitative integration of results and the assessment of statistical heterogeneity. Second, the included studies predominantly focused on healthy older adults, with relatively limited data on capillary adaptations in individuals with metabolic disorders or functional decline. Moreover, some studies concentrated solely on structural indices of capillarization (such as CD and C:F), lacking systematic evaluation of capillary function (e.g., substrate transport efficiency) and underlying molecular mechanisms, which restricts a comprehensive understanding of exercise induced capillary adaptations. Meanwhile, most of the included studies rely on two-dimensional histological indices, which to some extent limit the understanding of the structural complexity of the capillary network. Therefore, future research should expand the study population to include older individuals with higher risk profiles (e.g., those with sarcopenia) to enhance the real-world applicability of the findings. In addition, more comprehensive functional assessments of capillarization should be incorporated, along with 3D evaluation methods, to more fully capture the adaptive changes of the capillary network.

## 5 Conclusion

In summary, older adults still possess the ability to improve skeletal muscle capillarization through exercise. Aerobic exercise provides consistent angiogenic stimulation and promotes the expansion of the capillary network, whereas the adaptive effects of resistance training depend on an individual’s baseline capillarization status and muscle fiber type characteristics. Overall, improving capillarization indices and microcirculatory status through aerobic exercise can establish a favorable foundation. When capillarization levels are relatively high, resistance training can further enhance the structural and functional properties of skeletal muscle, thereby mitigating the adverse effects associated with skeletal muscle aging.

## Data Availability

The original contributions presented in the study are included in the article/Supplementary Material, further inquiries can be directed to the corresponding authors.
