# Supplementary material for: The role of exercise induced capillarization adaptations in skeletal muscle aging: a systematic review
Source: Front Physiol. 2025 Sep 26;16:1681184. doi: 10.3389/fphys.2025.1681184 (PMC12510980; doi:10.3389/fphys.2025.1681184)
Supplement: Supplementary file 1 [file Table1.docx]

Supplementary Material

# Supplementary File. Search Terms

Pubmed

(((((((Exercise[MeSH Major Topic]) AND (Skeletal muscle[MeSH Major Topic])) AND (Capillaries[MeSH Major Topic])) OR (Capillarization[Title/Abstract])) OR (Capillary Density[Title/Abstract])) OR (Capillary-to-Fiber Ratio[Title/Abstract])) OR (Capillary Supply[Title/Abstract])) AND (Aging[MeSH Major Topic])

Web of science

Results for (((TS=("Skeletal Muscle" OR "Skeletal Muscle Fiber" OR "Skeletal Muscle Fibers" OR "Myofiber" OR "Myofibers" OR "Skeletal Myofiber" OR "Skeletal Myofibers")) AND TS=("Capillarization" OR "Capillary Density" OR "Capillary-to-Fiber Ratio" OR "Capillary to Fiber Ratio" OR "Capillary Supply" OR "Capillary Contacts" OR "Capillary Contacts per Fiber" OR "Capillaries per Fiber" OR "Capillaries per Muscle Fiber")) AND TS=("Exercise" OR "Physical Activity" OR "Aerobic Training" OR "Resistance Training" OR "Endurance Training" OR "Combined Training" OR "Treadmill Running" OR "Wheel Running" OR "Voluntary Exercise" OR "Forced Exercise")) AND TS=("Aging" OR "Ageing" OR "Aged" OR "Older Adults" OR "Sarcopenia" OR "Age-related Muscle Decline" OR "Muscle Atrophy" OR "Age-related Muscle Loss")

Scopus

( TITLE-ABS-KEY ( "Exercise" OR "Physical Activity" OR "Aerobic Training" OR "Resistance Training" OR "Endurance Training" OR "Combined Training" ) ) AND ( TITLE-ABS-KEY ( "Capillarization" OR "Capillary Density" OR "Capillary-to-Fiber Ratio" OR "Capillary Supply" ) ) AND ( TITLE-ABS-KEY ( "Skeletal Muscle" OR "Skeletal Muscle Fiber" OR "Myofiber" ) ) AND ( TITLE-ABS-KEY ( "Aging" OR "Aged" OR "Sarcopenia" OR "Muscle Atrophy" ) ) AND ( LANGUAGE ( english ) ) AND ( PUBYEAR > 1999 AND PUBYEAR < 2026 ) AND PUBYEAR > 1999 AND PUBYEAR < 2026

Embase

('exercise'/exp OR 'physical activity' OR 'aerobic training' OR 'resistance training' OR 'endurance training' OR 'combined training') AND ('capillary'/exp OR 'capillarization' OR 'capillary density' OR 'capillary to fiber ratio' OR 'capillary supply') AND ('skeletal muscle'/exp OR 'skeletal muscle fiber' OR 'myofiber') AND ('aging'/exp OR 'aged' OR 'sarcopenia' OR 'muscle atrophy') AND [english]/lim AND [2000-2025]/py

# Supplementary Table. Study quality assessment results

| **Author, year** | **Q1** | **Q2** | **Q3** | **Q4** | **Q5** | **Score (5)** |
| --- | --- | --- | --- | --- | --- | --- |
| ***Quantitative randomized controlled trials*** | | | | | | |
|  | Is randomization appropriately performed? | Are the groups comparable at baseline? | Are there complete outcome data? | Are outcome assessors blinded to the intervention provided? | Did the participants adhere to the assigned intervention? |  |
| Leuchtmann, 2020 | Yes | Yes | Yes | Can’t tell | Yes | 4 |
| Mortensen, 2019 | Yes | Yes | Yes | Can’t tell | Yes | 4 |
| ***Non-randomized studies*** | | | | | | |
|  | Are the participants representative of the target population? | Are measurements appropriate regarding both the outcome and exposure? | Are there complete outcome data? | Are the confounders accounted for in the design and analysis? | During the study period, did the exposure occur as intended? |  |
| Charles, 2006 | Yes | Yes | Yes | Can’t tell | Yes | 4 |
| Croley, 2005 | Yes | Can’t tell | Yes | Can’t tell | Yes | 3 |
| Gavin, 2015 | Yes | Yes | Yes | Can’t tell | Yes | 4 |
| Gavin, 2007 | Yes | Yes | Yes | Can’t tell | Yes | 4 |
| Gliemann, 2014 | Yes | Yes | Yes | Can’t tell | Yes | 4 |
| Iversen, 2011 | Yes | Can’t tell | Yes | Yes | Yes | 4 |
| Moore, 2018 | Yes | Can’t tell | Yes | Can’t tell | Yes | 3 |
| Moro, 2019 | Yes | Yes | Yes | Yes | Yes | 5 |
| Murias, 2011 | Yes | Yes | No | Can’t tell | Yes | 3 |
| Olsen, 2020 | Yes | Yes | Can’t tell | Can’t tell | Yes | 3 |
| Prior, 2015 | Yes | Yes | Yes | Can’t tell | Yes | 4 |
| Ryan, 2006 | Yes | No | Yes | Can’t tell | Yes | 3 |
| Snijders, 2019 | Yes | Yes | Yes | Can’t tell | Yes | 4 |
| Snijders, 2017 | Yes | Yes | Yes | Yes | Yes | 5 |
| Verdijk, 2016 | Yes | Can’t tell | Yes | Can’t tell | Yes | 3 |
| ***Quantitative descriptive*** | | | | | | |
|  | Is the sampling strategy relevant to address the research question? | Is the sample representative of the target population? | Are the measurements appropriate? | Is the risk of nonresponse bias low? | Is the statistical analysis appropriate to answer the research question? |  |
| Betz, 2024 | Yes | Yes | Yes | Yes | Yes | 5 |
| Gliemann, 2021 | Yes | Can’t tell | Yes | Yes | Yes | 4 |
| McKendry, 2020 | Yes | Can’t tell | Yes | Yes | Yes | 4 |
| Pollock, 2018 | Yes | Can’t tell | Yes | Yes | Yes | 4 |
